# Non-inferior low-dose coronary computed tomography angiography image quality with knowledge-based iterative model reconstruction for overweight patients

**DOI:** 10.1371/journal.pone.0209243

**Published:** 2018-12-26

**Authors:** In Kyung Park, Jeffrey Park, Tae Hoon Kim, Joohee Lee, Kyunghwa Han, Chisuk Oh, Chul Hwan Park

**Affiliations:** 1 Department of Radiology and Research Institute of Radiological Science, Gangnam Severance Hospital, Yonsei University College of Medicine, Seoul, Republic of Korea; 2 College of Letters and Science, University of California Berkeley, Berkeley, CA, United States of America; 3 Department of Radiology and Research Institute of Radiological Science, Severance Hospital, Yonsei University College of Medicine, Seoul, Republic of Korea; Chongqing University, CHINA

## Abstract

We investigated the feasibility of low-dose coronary computed tomography angiography (CCTA), using a prospective electrocardiogram (ECG)-triggered axial scan protocol, knowledge-based iterative model reconstruction (IMR), and fixed tube current, in overweight subjects. Forty non-overweight (group A; body-mass index [BMI] < 25 kg/m^2^) and 40 overweight individuals (group B; BMI = 25–30 kg/m^2^), who underwent CCTA for coronary artery disease screening, were retrospectively and consecutively enrolled. A 64-slice CT scanner was used at 100-kVp tube voltage and 150-mA tube current, and images were reconstructed using IMR techniques. Image noise, attenuation at the aorta, signal-to-noise ratio (SNR), and contrast-to-noise ratio (CNR) at the proximal right and left main coronary arteries (pRCA and LMCA) were calculated. CCTA images were qualitatively evaluated using a four-point scale (1, poor; 4, excellent) and analyzed using a non-inferiority test with a pre-defined non-inferiority margin of -0.2. The mean CCTA radiation dose (Group A: 1.33 ± 0.02 mSv; Group B: 1.35 ± 0.10 mSv; *p* = 0.151) and mean aortic root CT attenuation values (Group A: 447.9 ± 81.6 HU; Group B: 439.5 ± 63.6 HU; *p* = 0.571) did not differ significantly between the two groups. The mean noise in groups A and B was 26.0 ± 4.8 HU and 29.2 ± 4.4 HU, respectively (*p* = 0.005). The noise reduction ratio in the groups, compared to filtered back projection, was 65.0% and 68.1%, respectively. The mean grade of image quality did not differ significantly (3.75 ± 0.04 vs. 3.71 ± 0.04, *p* = 0.478). Group B CCTA image quality was non-inferior (mean difference = -0.043, 95% CI = -0.162–0.077) to that of Group A. We concluded that low-dose CCTA with prospective ECG-triggering and IMR might be applied to overweight subjects, as well as to normal-weight subjects, by using a fixed tube current without an increase in tube current based on the patient’s body size.

## Introduction

Exposure to ionizing radiation is the main drawback of coronary computed tomography angiography (CCTA), which detracts from the advantages of CCTA, such as non-invasiveness and high diagnostic accuracy [1]. Reduction of the radiation dose of CCTA, while maintaining image quality, has been considered to be a challenging task. With technical advances in computed tomography (CT), various strategies have been implemented in a continuous effort to lower the radiation dosage of CCTA [2–5]. Among these, lowering tube voltage is an efficient strategy, because the radiation dose is proportional to the square of the tube voltage [2]. Currently, the Society of Cardiovascular Computed Tomography guidelines recommend using a tube voltage of 100 kV for patients < 30 kg/m^2^ [6]. When the tube voltage is decreased from 120 kVp to 100 kVp, about 58% reduction in radiation dose is possible [7]. Advanced reconstruction algorithms can be used to reduce the radiation dose without compromising image. The standard reconstruction technique is filtered back projection (FBP), which is fast, but leads to impaired image quality when lowering radiation dose [8]. Advanced iterative reconstruction algorithms, such as model-based iterative reconstruction (MBIR) or knowledge-based iterative model reconstruction (IMR), were introduced to overcome such limitations. These approaches attempt to identify the image that is the best fit to the original data, while reducing image noise. MBIR, which uses photon modeling, noise statistics, and system optics modeling, could reduce image noise up to 79%, as compared to FBP, however, the routine application of this technique is restricted by the long reconstruction times and limited user flexibility [9, 10]. In contrast, IMR, which uses a fully iterative algorithm and knowledge-based approach, can reduce reconstruction time and has become available for routine clinical practice. This algorithm can reduce image noise by up to 80% as compared with FBP in clinical CCTA studies [11]. Several reports have claimed that IMR could improve the image quality in low tube voltage CCTA in patients with a body mass index (BMI) < 30 kg/m^2^ [11, 12]. In addition, since radiation dose is proportional to the tube current [13], it is essential to optimize the tube current.

We assumed that with IMR, tube current increase according to body size might not be necessary, even if the subject is overweight and the tube voltage is 100 kVp, because IMR could reduce the image noise significantly. However, no previous report has investigated image quality and radiation dose in relation to BMI, using CCTA with IMR, a low tube voltage, and a fixed tube current. The purpose of this study was to evaluate the feasibility of using low-dose CCTA, implementing a prospective electrocardiogram (ECG)-triggered axial scan protocol, 100 kVp tube voltage, IMR, and fixed tube current, in overweight subjects.

## Material and methods

This study was reviewed and approved by the Institutional Review Board of our institution (Gangnam Severance Hospital; IRB number 3-2017-0023). Because this study was a retrospective observational study, the need for obtaining informed consent from participants was waived.

### Study population

The sample size was derived from preliminary image quality assessments of 10 subjects (5 with BMI < 25 kg/m^2^ and 5 with BMI = 25–30 kg/m^2^) who were eventually not included in this study. The margin of non-inferiority for the qualitative image quality, which served as the basis for the sample size calculation, was set as -0.2 [2]. The sample size calculations indicated a requirement of 40 subjects in each group, which allowed for a power of 90% and a two-sided α-level of 0.05, for demonstrating statistically significant evidence of the non-inferiority of this imaging approach in overweight subjects compared to non-overweight subjects.

Forty participants each from a non-overweight (group A; BMI < 25 kg/m^2^) and an overweight (group B; BMI = 25–30 kg/m^2^) group were enrolled retrospectively and consecutively; participants underwent CCTA for coronary artery disease screening using prospective ECG-gating CCTA. For subjects whose heart rates exceeded 65 bpm before examination, a β-blocker (25–50 mg propranolol hydrochloride; Pranol, Dae Woong, Seoul, Korea) was administered orally, 1 hour prior to CCTA. The exclusion criteria were as follows: (i) a heart rate exceeding 65 bpm even after oral administration of a β-blocker, (ii) arrhythmia, (iii) known hypersensitivity to contrast media containing iodine, (iv) decreased renal function (serum creatinine > 150 μmol/L), (v) hemodynamic instability, and (vi) congestive heart failure.

### Imaging protocol

All CT scans were obtained using a 64-slice CT scanner (Ingenuity Core 128, Philips Healthcare, Cleveland, Ohio, USA), in the craniocaudal direction, during a single breath-hold at end-inspiratory suspension. The scan range captured the heart from the carina level to the diaphragm. A step-and-shoot technique was used with a prospective ECG-gated protocol. The scanning parameters were as follows: (i) step-and-shoot axial scanning, (ii) 400-ms gantry rotation time, (iii) 100-kVp tube voltage, and (iv) 150-mAs tube current without a tube current increase based on the patient’s body size. Through an 18-gauge intravenous catheter placed in the antecubital fossa, Ioversol, containing a 350 mg/mL iodine solution (Optiray 350; Tyco Healthcare, Kantata, Canada), was injected at a rate of 4–5 mL/s. Thereafter, 50 mL of 0.9% saline was administered by a power injector (Dual Shot; Nemoto Kyorindo, Tokyo, Japan) at a speed of 5 mL/s. The body weight was used to determine the total dose of contrast (1 mL/kg). A real-time bolus-tracking method was used for imaging. The region of interest (ROI) was drawn at the proximal descending aorta. After 7 seconds, the scanning process proceeded only when the attenuation at the ROI exceeded 130 HU. In all scans, participants successfully executed the breath-hold maneuver. The subjects underwent simultaneous ECG recordings in each study. The dose-length product (DLP) was multiplied by 0.014 mSy / (mGy × cm), the conversion coefficient, in order to calculate the effective radiation dose [1, 14].

### CT image reconstruction

All CCTA images were reconstructed by knowledge-based iterative reconstruction (IMR-level 1; Philips Healthcare). The parameters for reconstruction were: (i) 0.9-mm slice thickness, (ii) 0.45-mm increments, (iii) 512 × 512-pixel image matrix, (iv) XCC kernel, and (v) 15–23-cm field of view. We fed the images through a picture archiving and communication system (PACS; Centricity 2.0, GE Medical Systems, Mt Prospect, IL, USA). Post-processing for CCTA was achieved using commercial software (Aquarius Workstation V3.6, TeraRecon, San Mateo, CA, USA).

### Quantitative analysis

Image quality was quantitatively analyzed in CCTA reconstructed with IMR in both non-overweight and overweight patients. On axial CT images, a round ROI was placed on the ascending aorta, proximal right coronary artery (RCA), and left main coronary artery (LM) to calculate the vascular attenuation values. To ensure that all three series of axial images were obtained at the same level, the cross-reference function on PACS was utilized. The image noise of CCTA was defined as the standard deviation of the attenuation values measured at the ascending aorta. The signal-to-noise ratio (SNR) and contrast-to-noise ratio (CNR) were calculated as follows.

$$\begin{array}{l} SNR=(vascular\;attenuation)/(image\;noise)\\ CNR=[(attenuation\;of\;vessel)‑(attenuation\;of\;the\;adjacent\;pervascular\;fat)]/(image\;noise) \end{array}$$

### Qualitative analysis

Two radiologists with 10 and more than 20 years of experience in cardiac CT, who were blinded to the patient’s medical records, independently performed qualitative assessment of the image quality of CCTA reconstructed with IMR in both groups A and B. They used a 4-point grading system at the four main coronary arteries (left main, left anterior descending, left circumflex, and right coronary artery) as follows [2,15].

Grade 1 (poor/non-diagnosable): severely degraded image, inability to evaluate vessel lumen
Grade 2 (adequate): moderately degraded image, minor difficulty in evaluating vessel lumen
Grade 3 (good): marginal image degradation, no difficulty in evaluating vessel lumen
Grade 4 (excellent): no detectable degradation of image

### Statistical analysis

Categorical variables were represented as numerical values of frequencies and/or percentages, while continuous variables were noted as mean ± standard deviation (SD). Data distribution was evaluated using the Shapiro–Wilk test and Q-Q plots. Demographic differences between the two allocated groups, such as age, height, weight, BMI, and heart rate, were analyzed using independent two-sample *t-*tests. Differences in sex distribution between two groups were assessed for statistical significance using a chi-square test. For analyzing differences between the groups in terms of CT attenuation, image noise, SNR, CNR, and radiation dose, independent two-sample *t-*tests were used. Interobserver reproducibility of CCTA attenuation and noise was verified by the Intraclass correlation coefficient (ICC). ICCs of <0.40, 0.40–0.75, and 0.76–1.00 indicated poor agreement, fair to good (moderate) agreement, and excellent agreement, respectively. Qualitative image quality was evaluated by using linear mixed model analysis, considering multiple vessels per patient. The 95% confidence interval (CI) was estimated to test the image quality differences between the two groups, qualitatively. The non-inferiority of the qualitative image-quality in group B compared to group A was demonstrated if the lower limit of the two-sided 95% CI lies above the non-inferiority margin. The non-inferiority margin for image quality differences among the two subject groups was set as -0.2 [2]. Interobserver agreement regarding the qualitative analysis of CCTA was evaluated using a linear-weighted Cohen’s kappa test. A kappa value of 0.00–0.20 signified none to slight agreement; 0.21–0.40, fair agreement; 0.41–0.60, moderate agreement; 0.61–0.80, good agreement; and 0.81–1.00, excellent agreement. All statistical analyses were performed using the Power Analysis and Sample-Size package (Version 12) and the SPSS 20 Statistical Package for the Social Sciences (Chicago, IL, U.S.A.).

## Results

### Interobserver agreement and data distribution

The overall data exhibited a normal distribution. Excellent interobserver reliability was proven in the quantitative analysis of CCTA image quality (ICC for attenuation = 0.998, ICC for noise = 0.845). Cohen’s kappa test showed good interobserver agreement in the qualitative assessment of the image quality of CCTA (mean kappa value = 0.768).

### Participant characteristics

Eighty patients (M:F 54:26, mean age 57.4 ± 9.6 years) who underwent CCTA for coronary artery disease screening were retrospectively enrolled. Forty individuals were allocated to group A (non-overweight group, BMI < 25 kg/m^2^) and another 40 were allocated to group B (overweight group, BMI ≥ 25 kg/m^2^). The mean radiation dose of CCTA was not significantly different between the two groups (1.33 ± 0.02 mSv vs. 1.35 ± 0.10 mSv, *p* = 0.151). The clinical characteristics of the two groups are summarized in Table 1. CCTA was performed without complications in all patients.

**Table 1:** Characteristics for 80 healthy adults underwent prospective electrocardiogram-gated coronary computed tomography angiography for screening.

Characteristics	Group A (non-overweight)	Group B (overweight)	*p*-value
**Number of subjects**	40	40	
**Age (years)**	58.0 ± 8.7	56.8 ± 10.6	0.575
**Male: Female**	23:17	31:9	0.094
**Height**	166.3 ± 8.6	167.6 ± 8.6	0.521
**Body weight (kg)**	64.5 ± 7.7	74.6 ± 7.9	<0.001
**Body mass index (kg/m^2^)**	23.2 ± 1.1	26.5 ± 1.0	<0.001
**Average heart rate (beats/min)**	54.7 ± 4.5	53.6 ± 4.7	0.650
**Effective radiation dose (mSv)**	1.33 ± 0.02	1.35 ± 0.10	0.151

All data are presented as the mean ± standard deviation

### Quantitative analysis

**Fig 1:**
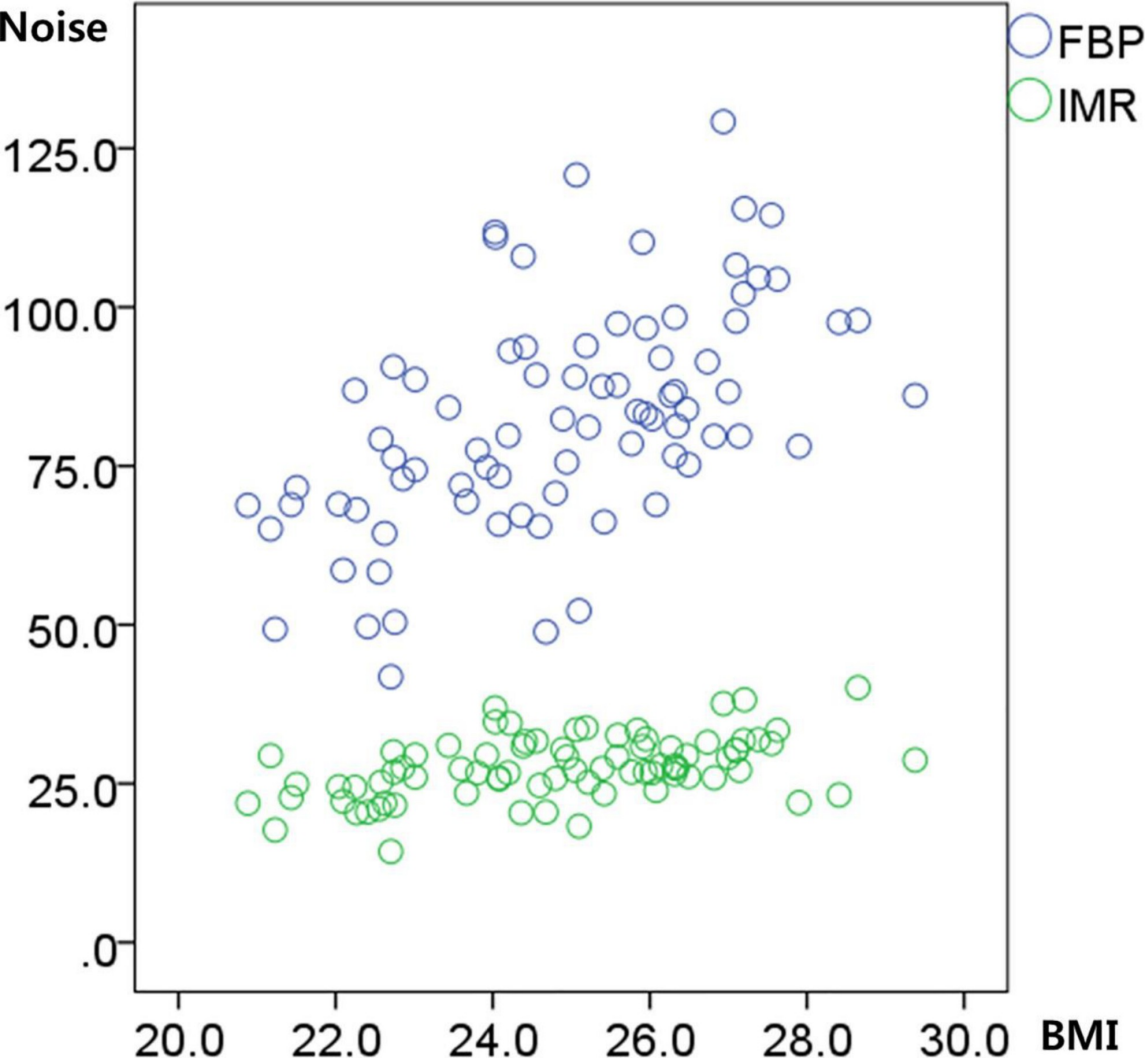
Image noise in CCTA with FBP reconstruction and IMR reconstruction. Image noise was calculated as the standard deviation of attenuation at the ascending aorta. The mean noise of 80 CCTA images were 82.5 ± 17.7 with FBP reconstruction, and 27.6 ± 4.8 with IMR reconstruction. The mean noise reduction ratio of IMR compared to FBP in 80 patients was 65.0% in Group A (non-overweight; BMI < 25 kg/m^2^) and 68.1% in Group B (overweight; BMI 25–30 kg/m^2^). BMI: body mass index. CCTA: coronary computed tomography angiography. IMR: iterative model reconstruction. FBP: filtered back projection.

The mean CT attenuation measured at the ascending aorta in CCTA did not differ between Group A and Group B (447.9 ± 81.6 HU vs. 439.5 ± 63.6 HU, respectively; *p* = 0.571). The mean noise in Group A was lower than that in Group B (26.0 ± 4.8 vs. 29.2 ± 4.4, respectively; *p* = 0.005). Group A showed significantly higher SNR at the RCA and LM than Group B (17.0 ± 4.4 vs. 15.2 ± 3.3, respectively; *p* = 0.044 in the RCA and 17.8 ± 4.6 vs. 15.4 ± 3.5, respectively; *p* = 0.013 in the LM). The CNR of the RCA and LM were also significantly higher in Group A than in Group B (20.9 ± 4.9 vs. 18.5 ± 3.6, respectively; *p* = 0.015 in the RCA and 22.0 ± 5.3 vs. 18.8 ± 3.8, respectively; *p* = 0.004 in the LM) (Table 2). The noise reduction ratio of IMR compared to FBP was 65.0% in Group A and 68.1% in Group B (Fig 1).

**Table 2:** Quantitative analysis of image qualities of coronary computed tomography angiography reconstructed with IMR in non-overweight and overweight groups.

		Group A (non-overweight)	Group B (overweight)	*p*-value
**Attenuation of the aortic root**		447.9 ± 81.6	439.5 ± 63.6	0.571
**Noise**		26.0 ± 4.8	29.2 ± 4.4	0.005
**SNR of the RCA**		17.0 ± 4.4	15.2 ± 3.3	0.044
**SNR of the LM**		17.8 ± 4.6	15.4 ± 3.5	0.013
**CNR of the RCA**		20.9 ± 4.9	18.5 ± 3.6	0.015
**CNR of the LM**		22.0 ± 5.3	18.8 ± 3.8	0.004

All data are presented as the mean ± standard deviation.

SNR: Signal-to-noise ratio, CNR: Contrast-to-noise ratio, RCA: Right coronary artery, LM: Left main coronary artery

### Qualitative analysis

**Fig 2:**
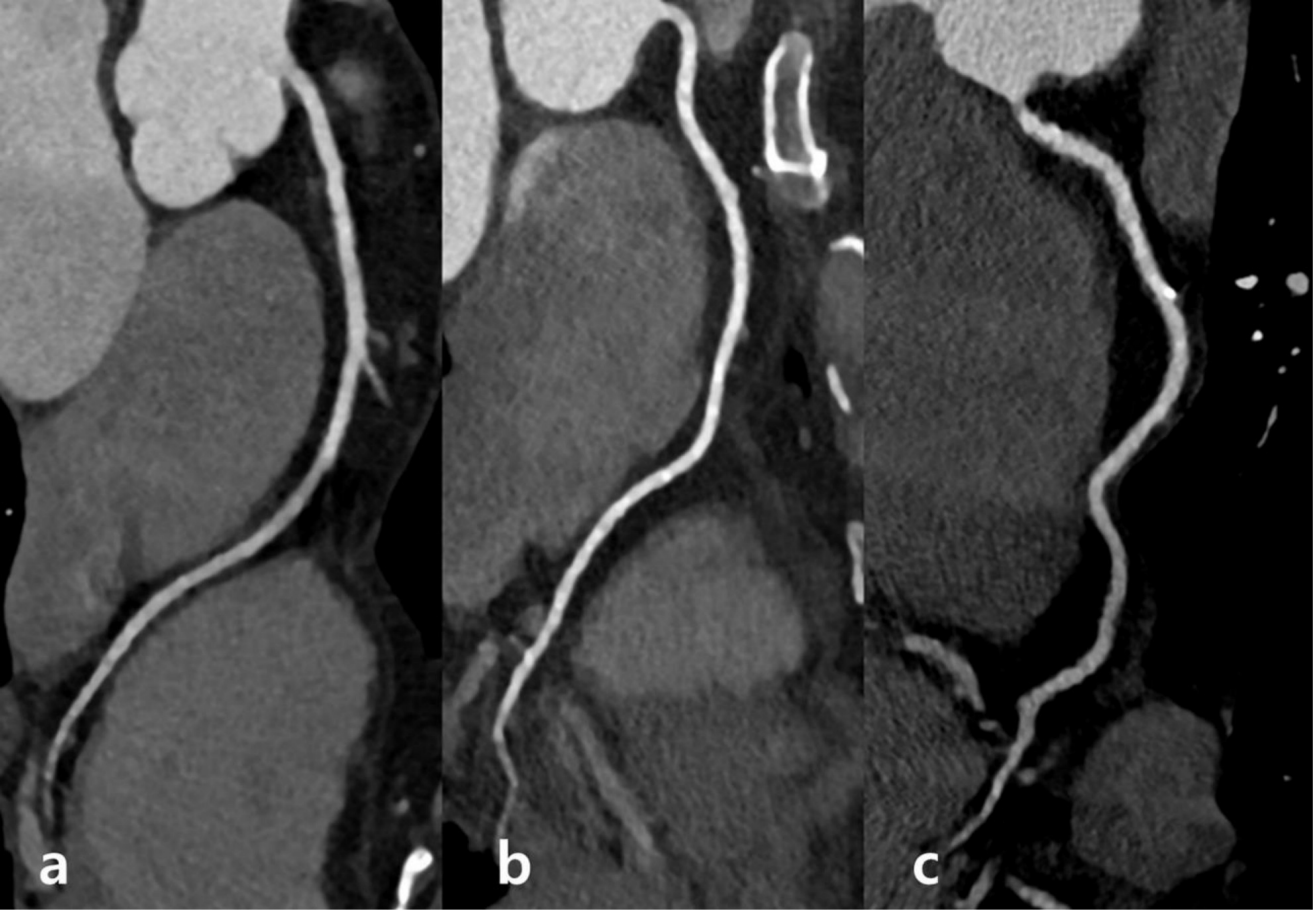
Representative CCTA Images with Different Image Reconstruction. Curved multiplanar images of the right coronary artery taken at 100 kVp, 150 mAs, with prospective ECG-triggering, and IMR reconstruction in subjects with BMI of (a) 22.1 kg/m^2^, (b) 27.5 kg/m^2^, and (c) 29.4 kg/m^2^. The images show good image quality with similar image noise and attenuation, irrespective of BMI. BMI: Body mass index. CCTA: coronary computed tomography angiography. ECG: electrocardiogram. FBP: filtered back projection. IMR: iterative model reconstruction.

The mean image quality of CCTA was 3.75 ± 0.04 in Group A, and 3.71 ± 0.04 in Group B (*p* = 0.478). The non-inferiority of the CCTA image quality in Group B was proven, as the lower limit of the 95% CI of the image quality difference exceeded -0.2, which is the pre-set non-inferiority margin (mean difference: -0.043, 95% CI: -0.162–0.077) (Table 3, Fig 2).

**Table 3:** Qualitative analysis of image qualities of coronary computed tomography angiography reconstructed with IMR in non-overweight and overweight groups.

	Group A (non-overweight)	Group B (overweight with IMR)	*p*-value
**Mean grade of four vessels**	3.75 ± 0.04	3.71 ± 0.04	0.478
**RCA**	3.63 ± 0.06	3.59 ± 0.06	0.629
**LM**	4.00 ± 0.00	4.00 ± 0.00	1.000
**LAD**	3.74 ± 0.06	3.65 ± 0.06	0.289
**LCX**	3.63 ± 0.06	3.60 ± 0.06	0.664

All data are presented as the mean ± standard error

RCA: Right coronary artery, LM: Left main coronary artery, LAD: Left anterior descending artery, LCX: Left circumflex artery

## Discussion and conclusion

Our study showed that CCTA with prospective ECG-triggering and IMR can be applied to overweight subjects as well as to normal weight subjects, using a fixed tube current, without the need for tube current modulation based on the patient’s body size.

In 2009, Hausleiter et al. [1] reported that the estimated radiation dose of CCTAs in 50 study sites was about 12 mSv, and emphasized the need for efforts to reduce radiation dosage. There are various strategies for reducing radiation dose, such as automatic exposure control, high-pitch helical imaging, tube current modulation, low tube voltage, and prospective ECG-gating, that can be applied in clinical practice [16–19]. Lowering tube voltage is effective for decreasing radiation dose in CCTA, given that the radiation dose is directly proportional to the square of the tube voltage [20]. Bischoff et al. [21] showed that using a 100-kV scan protocol could decrease 53% of the median radiation dose of CCTA as compared to the conventional 120-kV scan protocol, while maintaining the diagnostic image quality. Tube current is another major factor in determining overall radiation doses, and radiation dosage is directly proportional to the tube current [22]. In clinical practice, the tube current is usually adjusted according to the patient's body size [6].

With existing reconstruction based on FBP, the radiation dose needs to be doubled to attain the same level of image noise in patients with high BMI as for patients with standard BMIs [23]. Recently, advanced reconstruction methods have been developed and applied in daily practice, including IR, hybrid IR, knowledge-based IR or model-based IR, which could improve image quality with reduced radiation dosage [11, 24–25]. IMR is a systemic model-based approach combined with statistics, which decreases noise by the iterative minimization of the differences between acquired data and an ideal image [11, 13, 25].

In previous studies, we evaluated the feasibility of lowering tube voltage for CCTA in non-overweight patients, with BMI < 25 kg/m^2^, and showed that IMR reduced image noise in CCTA to 56–67%, as compared with FBP techniques [12, 15]. Oda et al. [13] reported that CCTA with 100 kVp and model-based type IR could improve qualitative and quantitative image quality; however, they used 100–300 mAs and the range of final radiation dosage varied from 0.9 mSv to 2.6 mSv, although they used the same tube voltage. We hypothesized that tube current modulation according to body size may not be necessary, because there is a square-root relationship between radiation dose and image noise [17], and IMR might compensate for the increase in image noise in overweight patients. In this study, the mean noise of CCTA differed between low and high BMI groups; however, the absolute difference was 3.2. The mean noise levels were less than 30, and the SNRs or CNRs of RCA or LM were higher than 15 in both groups, which was not lower than those reported in previous studies [11, 15]. On visual assessment, the qualitative image quality of CCTA with a fixed tube current was not significantly compromised in overweight patients as compared with non-overweight patients.

This study had a few limitations. First, IMR is one of many reconstruction algorithms and the results in this study could not be applied to other algorithms from other vendors, such as the ADMIRE and MBIR algorithms. Second, this was a single-center, retrospective study. Multi-center prospective clinical trials are needed to confirm our results. Third, we compared the quantitative or qualitative image qualities of CCTA, without evaluating the diagnostic accuracy for coronary artery disease. Fourth, non-inferiority test was conducted to compare qualitative image qualities. Statistical non-inferiority was found in a pre-defined non-inferiority margin, but clinical non-inferiority can not be guaranteed. Last, we did not perform a phantom study for spatial resolution assessment of our CT system. We focused on clinically evaluating the quantitative and qualitative image quality of CCTA with low radiation dose, based on previous reports [12, 15]. Future studies should include such a phantom study to validate our findings.

In conclusion, low-dose CCTA with 100 kVp, prospective ECG-triggering, and IMR might be applied to overweight subjects as well as to normal weight subjects, using a fixed tube current, without increasing tube current based on the patient’s body size.

## Supporting information

S1 File Attached files are data of 40 non-overweight (group A; body-mass index [BMI] < 25 kg/m2) and 40 overweight individuals (group B; BMI = 25–30 kg/m2), who underwent CCTA with prospective ECG-triggering and IMR for coronary artery disease screening.
(XLSX)
